# Development of remote radar based vital sign acquisition for emergency department patient triage

**DOI:** 10.3389/fmedt.2026.1759641

**Published:** 2026-06-10

**Authors:** Yossi Shaya, Gil Levy, Akiva Esterson, Tommer Stein, Alon Kurtsman, Henry Czerniak, Itai Gordon, Keren Hod, Pinchas Halpern, Sagi Zimilis, Debra West, Daniel Trotzky, Ilan Bublil, Michael J. Drescher

**Affiliations:** 1 Department of Emergency Medicine, Beilinson Hospital, Rabin Medical Center, Petah Tikva, Israel; 2 Faculty of Medical & Health Sciences, Tel Aviv University, Tel Aviv, Israel; 3 Department of Medical Innovation, Assuta Ashdod Medical Center, Ashdod, Israel; 4 Faculty of Health Sciences, Ben-Gurion University of The Negev, Be'er Sheva, Israel; 5 Basis Statistics, Jerusalem, Israel; 6 Health Care Israel, Ministry of Health Israel, Jerusalem, Israel; 7 ELTA System Ltd., Ashdod, Israel; 8 Department of Emergency Medicine, Assuta Ashdod Medical Center, Ashdod, Israel; 9 Medical Management, Tel Aviv Sourasky Medical Center, Tel Aviv, Israel; 10 Department of Epidemiology and Preventive Medicine, School of Public Health, Gray Faculty of Medical & Health Sciences, Tel Aviv University, Tel Aviv, Israel

**Keywords:** COVID-19, emergency department, innovation, SARS-CoV-2, triage, vital signs

## Abstract

**Introduction:**

To enable remote acquisition of patients' vital signs, ELTA Systems Ltd. developed a Radar remote vital signs acquisition system (TAMAR). The system identifies chest and neck respiratiory movements and pulse wave movements, enabling quantification of respiratory and pulse rates. The validation process of the TAMAR system is reported.

**Methods:**

A multicenter, prospective observational study was performed, enrolling low acuity patients who presented to 3 Emergency Departments. Patients provided consent and had their pulse and respiratory rates simultaneously measured by the TAMAR radar system and a traditional monitor (Mindray BeneVision N15). Vital signs were recorded continuously by both systems for 150 s, and later compared using Bland-Altman statistical analysis.

**Results:**

Between May and September 2021 we enrolled 315 patients. Measurements were calculated in 181/259 (70%) patients for Heart Rate (HR), and in 191/252 patients (76%) for Respiratory Rate (RR). For RR, the upper level of agreement (LOA) was 1.0, and the lower LOA was 1.6 respirations per minute. For HR, the upper level of agreement was 4.7, and the lower LOA was 3.5 beats per minute. All of these were well below the predetermined acceptable LOA of +/- 10 percent of the reference monitor rate.

**Discussion:**

The vital signs acquired by the TAMAR system, based on 90-second average measurements, are statistically adequate compared to traditional monitors, suggesting that TAMAR may replace traditional monitors for patients' triage.

## 1 Introduction

Emergency Departments (EDs) treat a wide variety of illnesses, from mild illnessess and minor injuries to life-threatening emergencies requiring immediate intervention. Patients in the ED are therefore triaged on arrival to determine the acuity of their complaint. This triage determination is meant to decide the order of priority in which patients are to be seen, and the amount of time patients can safely wait to see a Health Care Provider (HCP) (1, 2).

Triage is a complex process, often performed by an experienced nurse. Triage requires rapid integration of the patient's chief complaint, vital signs, symptoms, and medical history (1).

Infection prevention in the ED presents unique challenges (3, 4). Undifferentiated clinical presentations, variable patient acuity, frequent HCP-patient interactions, and simultaneous care of multiple patients all create obstacles to infection prevention practices (3).

The SARS-CoV-2 (COVID-19) epidemic presents further challenges to EDs, both to patient triage and to infection control. While most patients with COVID-19 experience mild to moderate symptoms and recover, some may have severe, life-threatening disease (5–14). Identification, triage, and stratification of suspected COVID-19 patients is therefore a priority, and several schemes have been suggested for these processes (15–17).

According to current evidence, the SARS-CoV-2 virus is spread and transmitted between people, primarily through respiratory droplets and direct contact routes (18–22). HCPs in contact with COVID-19 patients may therefore be at high risk of infection (23–27). Although the use of video consultation has been suggested as an alternative to some healthcare encounters (28, 29), ED triage usually requires the acquisition of vital signs (2), thereby mandating relatively close contact between an HCP and the patient. The ability to measure vital signs while eliminating the need for close contact between the HCP and the patient can prevent the spread of infectious diseases and provide safety to the HCP who is exposed to a large number of infected patients routinely. Thus, the ability to acquire the patient's vital signs remotely and avoid direct contact between the HCP and the patient can provide safety to the HCP, avoid protective measures in the initial encounter between the patient and the HCP, and streamline the triage process in the ED.

We therefore performed a multicenter prospective observational trial to assess the accuracy of vital signs obtained by traditional methods compared with those obtained by a novel remote vital-sign acquisition (RVSA) system. This system enables the remote acquisition of vital signs, with no direct contact between the sensors and the patient's body, and with no contact or even proximity of an HCP required.

## Materials and methods

The study was conducted between May-September 2021 in the EDs of three hospitals in Israel: The ED at the Beilinison campus of Rabin Medical Center, which according to pre-COVID (2019) data (30) treats approximately 102,700 patients per year; the ED of Shamir Medical Center, which treats approximately 131,400 patients per year; and the ED of Assuta Ashdod Medical Center which treats approximately 75,700 patients per year.

The study protocol was approved by Clalit Health Care Services (the largest Health Maintenance Organization in Israel) and by the ethics committees (IRB) at all three hospitals.

Patients presenting to the ambulatory-care wing of the ED during the study period were approached by a researcher or by a research assistant, after being triaged to a non-urgent category by the on-shift triage nurse, and asked to participate in the study. Exclusion criteria included patients requiring urgent medical attention, as assessed by the triage nurse or by the researchers; patients under the age of 18; pregnant patients; patients with electronic devices such as cardiac pacemakers or insulin syringe pumps or with large metals of any kind on their body; and patients unable to give informed consent, either due to mental states, language barriers, or any other reason.

**Figure 1:**
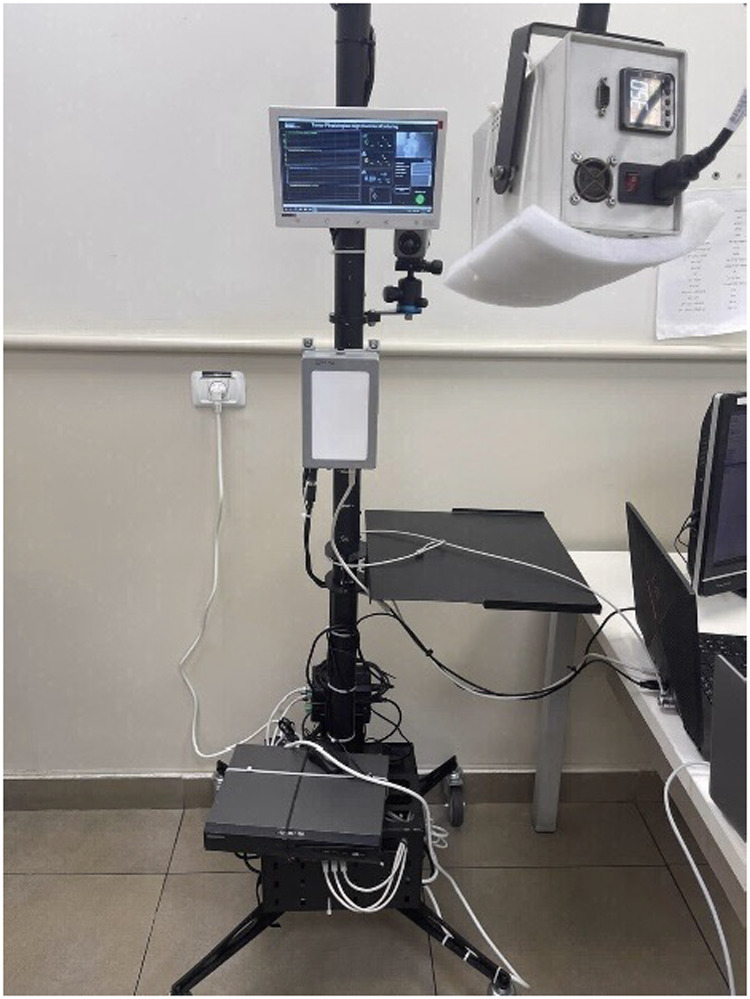
Tamar prototype.

Patients who provided oral and written consent t had their vital signs recorded by the novel RVSA system (TAMAR by ELTA Systems LTD) while simultaneously being monitored by traditional monitoring equipment (Mindray BeneVision N15). (Figure 1) The TAMAR Radar parameters include a frequency band of 24.0–24.24 GHz, and Emitting power with Antenna of 26 dBm.(Appendix A) The reference heart rate (HR) was acquired using Mindray's SpO2 finger sensor, and the reference respiratory rate (RR) was acquired using a CO2 Nasal sample cannula.

The patient was asked to sit 1–1.5 meters in front of the TAMAR, and vital signs were continuously recorded by both systems for 150 s. The distance between the TAMAR and the patient remained constant and was not considered a factor in measurement accuracy. Additionally, no restrictions were imposed regarding clothing thickness or room lighting conditions, as the radar technology is expected to be unaffected by these factors and thus applicable to real-life triage settings. A technician was present during measurements; however, positioning was controlled to minimize interference. The technician remained outside the primary sensing axis and at a greater distance than the patient. This setup reflects realistic clinical deployment conditions and supports the robustness of the system in non-isolated environments. No measurable signal interference attributable to the technician was observed. Due mainly to limitations of the reference system (the Mindray monitor), the initial 60 s were discarded, and only measurements taken during the following 90 s were used. To evaluate agreement between two measurements of RR and HR from two systems, the measurements were compared using Bland-Altman (mean difference or limits of agreement) analysis (31).

Radar signal processing and vital sign extraction were performed using a multi-stage pipeline designed to isolate physiological micro-motions from background noise and motion artifacts. The raw radar signal, acquired in the 24 GHz band, was first converted into a baseband signal representing phase variations associated with chest wall displacement. The signal was decomposed into respiratory and cardiac components using band-pass filtering. Based on known physiological frequency ranges in adults, respiratory signals were extracted using a band-pass filter of approximately 0.1–0.5 Hz (6–30 breaths/min), while cardiac signals were isolated using a higher-frequency band-pass filter of approximately 0.8–2.5 Hz (48–150 beats/min). These ranges were selected to encompass expected values in an ED population while minimizing overlap and noise contamination. To reduce the impact of gross body movement and environmental interference, several preprocessing steps were applied. First, a high-pass filter was used to remove low-frequency drift unrelated to physiological motion. Second, segments with excessive signal variance or abrupt amplitude changes, indicative of motion artifacts, were automatically flagged and excluded. In addition, the radar system incorporates proprietary clutter-reduction algorithms that suppress static reflections from the environment and isolate dynamic signals originating from the patient. Data exclusion was based on predefined signal quality thresholds and acquisition integrity criteria, consistent with early-stage device validation studies. Given the multi-site nature of the study and the investigational status of the system, strict filtering was required to ensure analytical validity. The differing sample sizes for HR and RR reflect modality-specific signal robustness, which is expected in early-stage contactless sensing technologies and is addressed as a limitation in the Discussion. Measurements were discarded if motion artifacts exceeded predefined thresholds, as described in the exclusion criteria. Following filtering, the processed signals were analyzed to extract periodicity. For respiratory rate (RR), peak detection was performed on the respiratory waveform using a zero-crossing and local maxima detection approach, ensuring temporal consistency between successive cycles. For heart rate (HR), due to the lower signal amplitude and higher susceptibility to noise, a combination of envelope detection and spectral analysis (fast Fourier transform, FFT) was used to identify the dominant frequency component within the cardiac band. The final HR and RR values were calculated as the average rate over the analyzed 90-second window, with one estimate per second aggregated into a mean value for comparison with the reference monitor. Signal quality indices were continuously computed, and only segments meeting predefined signal-to-noise and stability criteria were included in the final analysis. This approach ensured that extracted HR and RR values reflected reliable physiological signals rather than noise or artifact.

We used the classical Bland-Altman design to evaluate the comparison between TAMAR and the Mindray monitor. In this design, each of the two measurement methods is measured once on each patient at nearly the same point in time. The result of each method was calculated by the arithmetic average of the recordings during the last 90 s (one recording per second).

Based on accepted HR monitors standards (32), we predefined a minimum LOA for HR of no greater than ±10 percent of the reference rate or ±5 beats/minute, whichever was greater. For RR, we predefined a LOA of ±4 breaths/minute. These limits allowed us to set criteria for theaccuracy of measurements between the TAMAR and the traditional monitors. To complement agreement analysis, we quantified pointwise error between the Mindray (test) and Tamar (reference) RR measurements using standard metrics: Mean Absolute Error (MAE), Root Mean Square Error (RMSE) and Mean Absolute Percentage Error (MAPE). These metrics provide complementary information to Bland–Altman analysis by quantifying absolute, squared, and relative deviations, respectively. To assess signal robustness, respiratory signals were analyzed in the frequency domain. Power spectral density (PSD) was estimated (e.g., Welch's method) to identify the dominant respiratory frequency band and evaluate spectral concentration. Additionally, a time–frequency representation (e.g., short-time Fourier transform, STFT) was used to examine temporal stability of the respiratory component under varying physiological conditions. Signal quality was assessed based on: Stability of the dominant respiratory peak, Signal-to-noise characteristics within the respiratory band and Temporal continuity of frequency components. This approach is consistent with recent studies demonstrating that combining time-domain validation (e.g., Bland–Altman, RMSE) with spectral analysis enhances robustness assessment of respiratory monitoring systems (33).

The predefined LOA were selected to reflect clinically acceptable variability in ED triage decision-making rather than purely statistical thresholds. For HR, the criterion of ±10% or ±5 beats/min (whichever is greater) aligns with widely accepted performance standards for bedside monitoring devices and reflects the tolerance within which HR variability does not alter triage categorization or immediate clinical management. For example, in adult ED triage systems (e.g., ESI), HR thresholds that influence acuity are typically separated by margins exceeding 10–15 beats/min; therefore, deviations within ±5 beats/min are unlikely to result in clinically meaningful misclassification. For RR, the predefined LOA of ±4 breaths/min was selected based on its role as a sensitive but variable vital sign in acute care. Clinical thresholds for tachypnea (e.g., ≥20–22 breaths/min in adults) are sufficiently spaced such that a ± 4 breaths/min deviation would rarely shift a patient across critical decision thresholds (e.g., normal vs. clinically significant tachypnea) in stable, non-urgent populations. Moreover, RR measurement by standard reference methods (capnography or manual counting) is itself subject to intra- and inter-observer variability of a similar magnitude, supporting the clinical appropriateness of this margin. Importantly, these LOA thresholds were defined to ensure that agreement between the radar-based system and conventional monitoring would preserve clinical equivalence in triage, rather than requiring exact numerical concordance. To further evaluate system performance across clinically relevant physiological ranges, a predefined subgroup analysis was conducted. Measurements were stratified according to HR and RR categories reflecting clinically meaningful thresholds in ED practice: HR (<60, 60–100, >100 beats/min) and RR (<12, 12–20, >20 breaths/min). Bland–Altman analysis was repeated within each subgroup to assess whether agreement between the radar-based system and the reference monitor was maintained across normal and abnormal physiological states. A sample size of 200 was calculated based on a power of 80% and a statistical significance of 5%. In other words, power is the probability that you will reject the null hypothesis when you should (and thus avoid a Type II error). That is, you should have an 80% or greater chance of finding a statistically significant difference when there is one.

**Figure 2:**
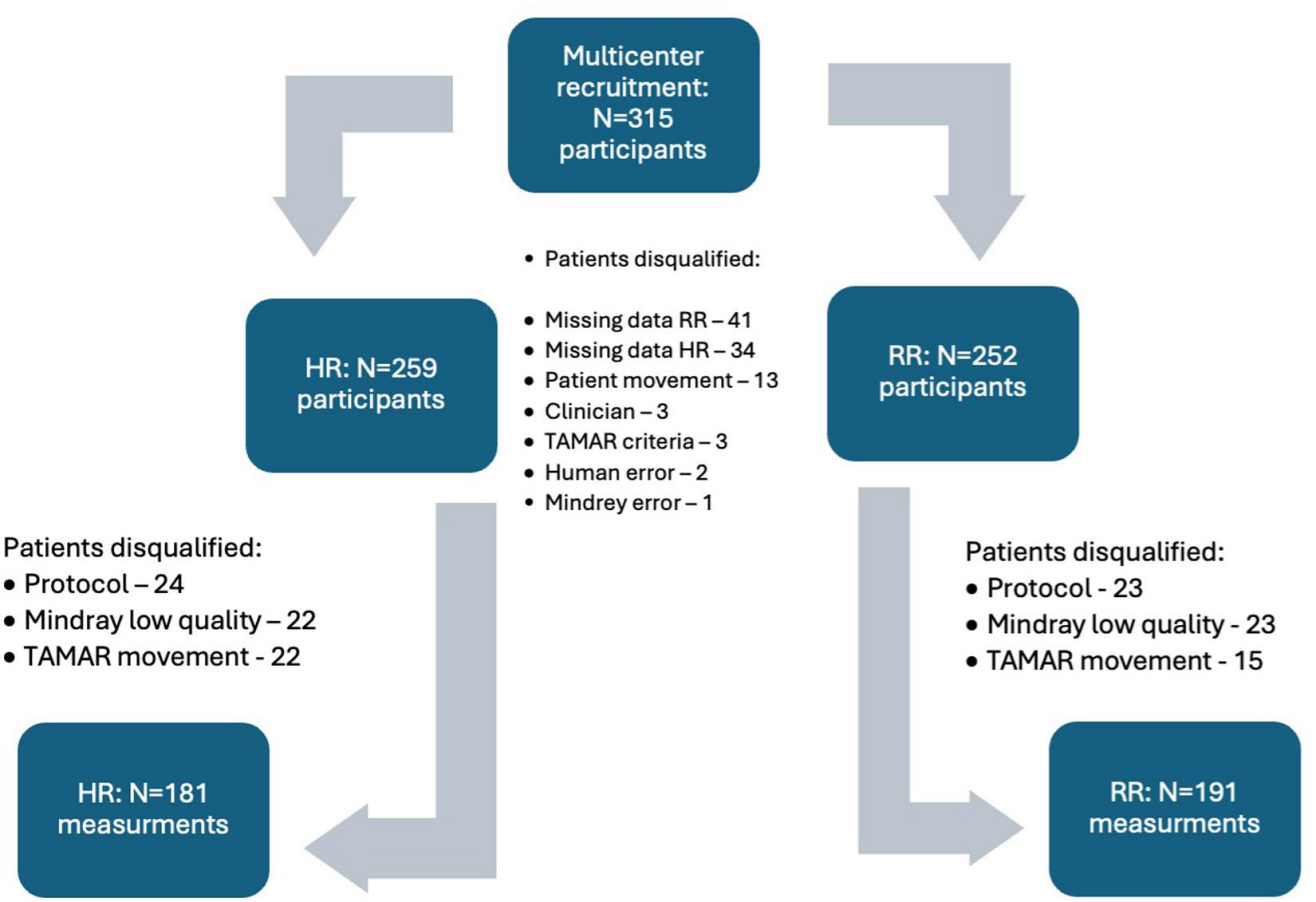
Excluded patient during data analysis. HR, heart rate; RR, respiratory rate.

Measurements were disqualified if meta-data was unrecorded whether due to technical or operator error, if the data recorded by the reference system was deemed unreliable by an unbiased and blinded technician of the reference system (for example if the O2 saturation monitor Pulse Index was below 0.5, or if drastic changes in RR were recorded) if time synchronization between the two systems was lost if the patient moved during the measurement (as reported either by the operator or by the radar system), or if another person entered the testing field of the radar system during the measurement (Figure 2).

## 3 Results

Between May and September of 2021, we recruited 315 patients in 3 EDs (113 in Belinson, 110 in Shamir, and 92 in Assuta). Demographic data is presented in Table 1. For the RR analysis, due to predefined data quality criteria and technical acquisition limitations (e.g., inadequate signal quality or incomplete recordings), 124 patients were excluded (see Figure 2), leaving 191 patients in the final analysis. Similarly, 134 patients were excluded, from the HR analysis, leaving 181 patients in the final analysis (Figure 2).

**Table 1:** Demographic data.

Characteristic	Heart rate	Respiratory rate
N, Male	101	102
N, Female	65	72
Age (mean ± SD), Male	46 +/- 18	44 +/- 18
Age (mean ± SD), Female	42 +/-16	40 +/- 15
Height (mean ± SD), Male	173 +/- 8	173 +/−9
Height (mean ± SD), Female	167 +/- 8	167 +/- 9
Weight (mean ± SD), Male	80 +/- 16	79 +/- 17
Weight (mean ± SD), Female	75 +/- 20	75 +/- 20

For the RR, Bland-Altman analysis demonstrated a mean bias of −0.43 ± 1.17 breaths/min, indicating minimal systematic difference between the two measurement methods. The 95% LOA ranged from −2.74 to 1.87 breaths/min, with no evidence of proportional bias across the measurement range. (Table 2) Confidence intervals: Mean bias 95% CI: −0.51 to −0.35 breaths/min, Lower LOA 95% CI: −2.95 to −2.53 breaths/min, Upper LOA 95% CI: 1.66 to 2.08 breaths/min. A strong linear association was observed between Mindray and TAMAR RR measurements (Pearson's r = 0.967, *p* < 0.001). Lin's concordance correlation coefficient (CCC) demonstrated excellent agreement (CCC = 0.965). With *n* = 191, the 95% CI the CCC = 0.965 (95% CI: 0.952–0.975). This demonstrate “excellent agreement” range (>0.90). Agreement within predefined clinical thresholds: ≥99% of measurements within ±4 breaths/min and ∼96%–98% within ±10% of reference values. For HR, Paired Bland-Altman analysis demonstrated a small mean bias of 0.50 ± 2.09 bpm, indicating minimal systematic difference between the two measurement systems. The 95% LOA ranged from −3.58 to +4.59 bpm, reflecting a relatively narrow dispersion of differences. (Table 3) The 95% confidence intervals (CI) for the limits of agreement were: Lower LOA: −4.01 to −3.15 bpm and Upper LOA: +4.16 to +5.02 bpm. A strong linear relationship between the two systems was observed, with a Pearson correlation coefficient (r) of 0.990. To further assess agreement beyond correlation alone, Lin's concordance correlation coefficient (CCC) was calculated, yielding a value of 0.986, indicating near-perfect concordance by accounting for both precision and accuracy. To evaluate clinical performance, predefined acceptance thresholds were applied: ±5 bpm: 96.0% of measurements fell within this range and ±10% relative error: 97.8% of measurements were within this threshold. These findings indicate that the vast majority of measurements meet commonly accepted criteria for clinical accuracy in HR monitoring. Overall, the TAMAR system demonstrated high agreement, strong correlation, and excellent concordance with the reference device. The small bias, narrow limits of agreement, and high proportion of measurements within clinically acceptable thresholds support the system's statistical and clinical adequacy for HR assessment in the studied population. (Table 3)Regards the respiratory rate data, Error-based analysis demonstrated low deviation between methods: RMSE: 1.25 breaths/min, MAE: 1.0 breaths/min and MAPE: ∼5%–7%**.** These values indicate high accuracy and are consistent with performance reported in comparable respiratory monitoring systems. Frequency-domain analysis demonstrated a well-defined dominant spectral peak within the physiological respiratory band across recordings, with minimal spectral dispersion. The peak frequency corresponding to respiratory rate remained stable across time and between measurement systems. Time–frequency analysis further showed consistent temporal tracking of respiratory frequency, without significant drift or fragmentation, supporting robustness of signal extraction under varying conditions. The preservation of a stable respiratory spectral signature, together with low RMSE and narrow limits of agreement, indicates that the system maintains high signal fidelity and robustness. These findings are consistent with recent reports highlighting the importance of combined time-domain and spectral validation in respiratory monitoring technologies (33). The integration of time-domain accuracy metrics with frequency-domain validation provides complementary evidence of both measurement agreement and signal robustness, as recommended in recent respiratory sensing studies.

**Table 2:** Bland-Altman analysis of respiratory rate (RR). Comparing the upper and lower levels of agreement and 95% conf. intervals.

Parameter	Estimate (breaths/min)	95% Confidence Interval (breaths/min)
Mean bias	−0.43	−0.51 to −0.35
Lower LOA	−2.74	−2.95 to −2.53
Upper LOA	1.87	1.66 o 2.08

Precentage of points within ±10% (±4 breaths/min): 99.0% of measurements.

**Table 3:** Bland-Altman analysis of heart rate (HR). Comparing the upper and lower levels of agreement and 95% conf. intervals.

Parameter	Estimate (beats/min)	95% Confidence Interval (beats/min)
Mean bias	0.50 bpm	0.2–0.8
Lower LOA	−3.58	−4.01 to −3.15
Upper LOA	4.59	4.16 o 5.02

Percentage of points within ±10% (±5 bpm): 96.0% of measurements.

Regards to the heart rate data, Bland–Altman analysis demonstrated a mean bias of 0.50 bpm (95% CI: 0.20 to 0.80 bpm), indicating a minimal systematic difference between the Tamar device and the reference system. The 95% limits of agreement (LOA) ranged from −3.5 to 4.7 bpm (95% CI: −4.0 to −3.5 bpm for the lower LOA and 4.7 to 5.2 bpm for the upper LoA), reflecting a narrow dispersion of differences across the physiological HR range. Correlation and concordance, a strong linear association was observed between the two systems (Pearson's r = 0.96), with similarly high agreement demonstrated by Lin's concordance correlation coefficient (CCC = 0.95), confirming both precision and accuracy of the measurements.

To complement agreement analysis, additional performance metrics were calculated. The RMSE was 2.14 bpm, the MAE was 1.14 bpm, and the MAPE was 1.45%, indicating low absolute and relative error. From a clinical perspective, 96.0% of measurements were within ±5 bpm, and 97.8% were within ±10% of the reference HR values, supporting high clinical acceptability of the system.

Exploratory subgroup analysis suggested consistent agreement across clinically relevant HR and RR ranges, including patients with elevated HR and RR; however, these findings should be interpreted cautiously given the limited sample size in extreme physiological ranges.

## Discussion

The first report of radar-based vital sign detection was a simple respiration monitoring of a cat in 1975 (34). Since then, the feasibility and accuracy of radar-based systems for human vital sign monitoring have been widely studied (35–48). These systems generally rely on the measurement of chest wall movement with respiration and the detection of the vibrations on the human skin affected by heartbeats. The use of such systems has been suggested for the detection of simulated earthquake victims (49, 50), and for the monitoring of patients with infectious organisms or under biochemical hazard conditions (51–53). They have mostly been tested in laboratory settings and on healthy volunteers, although recently a few clinical trials have been published in awake postoperative settings (54) and in sleep laboratories (55). However, to the best of our knowledge, our current report is the first demonstration of clinical ED use of such a system.

Radar-based vital sign measurements eliminate the need for direct contact between a potentially contagious patient and the HCP. Furthermore, since there is no contact between the patient and the measurement equipment, disinfection of the equipment between patients is no longer needed.

We have shown that the vital signs acquired by the radar-based TAMAR system are adequately comparable to those of the traditional monitor.

For HR, we predefined a minimum LOA of no greater than ±10 percent of the reference rate or ±5 beats/minute, whichever was greater, based on an accepted standard for cardiac monitors (32). We found thatTAMAR's measurements were well below the ±10 percent requirement of the reference rate (Table 3).

For RR, we predefined a LOA of ±4 breaths/minute. While other vital signs, such as HR, are measured objectively using automated technology, the ‘gold' standard for measuring RR has been to visually observe the chest and manually count breaths. Several studies have shown large differences between RR measured by this ‘gold' standard and objective measurements, up to 4.1 breaths/minute for RR measurements made sequentially by the same observer, and 4.3 breaths/minute for simultaneous measurements made by different observers (56–60). As we were assessing the accuracy of TAMAR as a triage tool, not as a monitor, we set our standards to the current ‘gold' standard. Eventually, we found that TAMAR's measurements were below this requirement (Table 2).

The present analysis demonstrates that the Tamar system provides accurate and reliable HR measurements when compared to a reference device, with minimal bias and narrow limits of agreement. The observed mean bias (<1 bpm) is statistically significant but clinically negligible, supporting the practical equivalence of the two systems. Importantly, this study extends beyond traditional Bland–Altman analysis by incorporating complementary performance metrics, including RMSE, MAE, and MAPE. These metrics quantify absolute and relative error and provide a more comprehensive characterization of measurement performance. The low values observed across all metrics indicate that measurement discrepancies are minimal and consistent across the HR range. Furthermore, the high percentage of measurements within predefined clinical thresholds (±5 bpm and ±10%) strengthens the evidence for clinical usability, as such thresholds are commonly used in validation studies of physiological monitoring technologies.

Recent advances in biosensor validation emphasize the importance of multidimensional analytical frameworks, combining agreement statistics with error metrics and signal-quality assessment. In particular, contemporary studies highlight the role of advanced signal-processing approaches, including structured decomposition models and multidomain analysis, in improving robustness and interpretability of physiological signals (33). While the present study focused on time-domain agreement and error analysis, we incorporated frequency-domain and time–frequency analyses to further evaluate signal robustness under motion, noise, and varying physiological conditions. Such approaches could provide additional insight into system performance in real-world and remote monitoring scenarios. Overall, the combination of strong agreement, low error metrics, and high clinical accuracy supports the conclusion that the Tamar system demonstrates statistically robust and clinically acceptable performance for heart rate monitoring.

Although the proportion of missing data (30%) in our study was relatively high (primarily due to technical computer-related issues rather than limitations of the technology itself), the sample size evaluated in this system remains substantially larger than that reported for similar systems described in the literature (54, 55, 61, 62).

Currently, RR is often not measured by nursing staff at all (59, 63). Although not directly assessed in the current study, we believe that the availability of an objective, non-invasive, simple tool for RR assessment may revolutionize the reliability of RR measurements at triage. Furthermore, radar technology appears to have the potential to quickly and easily acquire more detailed information about patients' breathing patterns, such as Inspiratory:Expiratory ratio (I:E ratio) and more, bringing data to the bedside that, until now, required spirometry. The reliability of these measurements will be compared with that of classic spirometry in future studies.

While currently only assessed in the ED triage setting, we believe TAMAR may be valuable in many other settings, including but not limited to: prolonged monitoring of bedridden patients as an alternative to classic monitors, monitoring of a group of patients ambulatory within a predefined area, monitoring of patients during ambulance or helicopter transport, and more.

Various alternative technologies are being studied for the contactless assessment of vital signs, including thermal imaging (64), video cameras (65, 66), and laser doppler (67, 68). Future studies will have to compare the accuracy, advantages, and disadvantages of the various technologies mentioned.

In recent years, contactless vital sign monitoring has advanced substantially, encompassing radar-based systems, camera-based remote photoplethysmography (rPPG), thermal imaging, and multimodal sensing approaches. Contemporary studies demonstrate improving accuracy for heart rate estimation using camera-based systems, although performance remains sensitive to motion artifacts, ambient lighting conditions, and patient-specific factors such as skin tone and perfusion variability (69–72). Thermal imaging approaches have shown feasibility for respiratory monitoring and fever screening but are limited by environmental dependency and lower temporal resolution (73, 74). Within radar-based technologies, recent work has focused on enhanced signal processing, motion compensation algorithms, and extraction of more complex physiological features, including cardiopulmonary coupling and preliminary waveform reconstruction; however, these studies are still largely confined to controlled laboratory environments, sleep laboratories, or small inpatient cohorts, often using fixed or bed-integrated configurations rather than mobile systems (75–78).

Against this evolving landscape, the present study contributes by demonstrating the feasibility of a mobile radar-based platform deployed within a real-world emergency department triage workflow, characterized by high patient turnover, environmental variability, and minimal preparation time. In contrast to many recent systems that emphasize signal richness or multimodal fusion, the TAMAR system is designed with a focus on clinical practicality, rapid acquisition, and infection-control advantages, with validation centered on rate-based outputs directly relevant to triage decision-making. This positions the system within an emerging class of workflow-integrated, point-of-care contactless monitoring technologies, bridging the gap between experimental capability and routine clinical implementation. Importantly, while radar sensing inherently captures displacement signals that may enable waveform-level analysis, the current study validates rate extraction only, and further work is required to assess the accuracy and clinical utility of derived waveform features against established reference standards. Currently, there are no widely adopted, regulator-approved radar-based systems integrated into emergency department triage workflows for contactless vital sign monitoring. Existing technologies in the broader contactless monitoring domain include: Camera-based remote photoplethysmography (rPPG) systems or Doppler radar prototypes evaluated in controlled environments. However, these systems are generally at earlier technology readiness levels (TRLs) or have been validated in non-acute settings (e.g., sleep laboratories, home monitoring), with limited evidence in high-throughput, real-world ED environments. Importantly: TAMAR is not currently marketed and has not undergone formal regulatory approval (e.g., FDA clearance or CE marking). The current study represents a clinical feasibility and validation phase. Direct commercial comparison is therefore not applicable at this stage.

From a regulatory and innovation perspective, TAMAR represents a system-level integration and clinical application innovation, rather than a fundamentally novel sensing modality. As such, its value proposition lies in clinical workflow integration and real-world deployment rather than proprietary sensing physics.

### Limitations

The current study was a pilot, proof-of-concept study conducted in the ED ambulatory wings. The patients enrolled in the study were triaged to low acuity, and therefore most participants (though not all) had vital signs within the normal range. A follow-up study is currently underway in ED zones of higher acuity and in other settings to assessthe systems' accuracy in assessing vital signs outside of the normal range.

Patients in this study were monitored for 150 s and required to sit still for the duration of the measurement. However, this was done primarily to obtain a large number of data points for comparison, not as a technical requirement of the system. The system can display measurements within a few seconds of the patient sitting in front of the sensor. On the other hand, a follow-up study is currently being planned to assess the system's ability to perform prolonged patient monitoring, rather than point measurements. To these ends, the system's software will be upgraded to either filter out patient movements or, at a minimum, flag measurements acquired during patient movements as less reliable, so that the clinician may take this information into account.

Researchers in the current study were unblinded topatients' vital signs, which were displayed in real time on the monitor screen. However, since the data were automatically recorded from both systems,later assessed for completeness by unrelated technicians, andthen analyzed by a third-party statistician, we believe this did not affect the results.

The system in our study currently lacks the ability to assess some traditional vital signs, such as Blood Oxygen Saturation (SaO2), Blood Pressure (BP), and body temperature, despite their importance as a central component of vital signs assessment at the triage station; however, this feature of body tempearture is currently under development. While BP is not part of commonly used triage systems such as the Emergency Severity Index (ESI) (2), and O2 Sat is not part of other commonly used scores such as the Modified Early Warning Score (MEWS) (79) or the Quick Sequential Organ Failure Assessment (qSOFA) Score (80), research is underway to assess for the ability of the systems to provide these measurements, and/or to provide other respiratory and circulatory information that may prove equally effective in triage (such as assessment of Tidal Volume, Cardiac Output, Compensatory Reserve (81, 82), and fetal heart monitoring. Previous reports have described the use of remote monitoring based on radar technology in clinical settings. Hoang Thi Yen et al. (61) reported only 10 individuals, and Fabian Michler et al. (62) reported on a device located under the hospitalized patient's bed. Our report is the first one to describe the mobile use of Radar technology in an ED setting with a large volume of patients in a multi-center study. The system is still under development, with planned additional features to better support real-world clinical workflows and triage needs. The present study was intentionally scoped as a technical and clinical validation study, focusing on measurement accuracy and feasibility. While the anticipated benefits—such as infection control, reduced staff exposure, and improved workflow efficiency—are highly relevant, formal assessment of: clinical outcomes, patient-reported outcomes and health-economic impact requires prospective implementation studies and regulatory-grade clinical trials, which are planned as future phases of development.

## Conclusions

Based on 90-second average calculations, the TAMAR system can reliably and remotely acquire HR and RR in low-acuity ED patients without physical contact with a healthcare provider and may potentially replace traditional methods for acquiring these measurements. Further research is required to assess TAMAR's reliability in other clinical settings, its ability to monitor patients over prolonged periods, and its comparability with alternative remote monitoring technologies.

## Data Availability

The datasets presented in this article are not readily available because the datasets presented in this article are not readily available of ethical and regulatory restrictions. Requests to access the datasets should be directed to corresponding author GL upon reasonable request and subject to appropriate ethical approvals. Requests to access the datasets should be directed to 2gillevy@gmail.com.
