# Supplementary material for: Development of remote radar based vital sign acquisition for emergency department patient triage
Source: Front Med Technol. 2026 Jun 10;8:1759641. doi: 10.3389/fmedt.2026.1759641 (PMC13290987; doi:10.3389/fmedt.2026.1759641)
Supplement: Supplementary file 1 [file Datasheet1.pdf]

TAMAR Technical Specifications

**Power**	
External Power Supply	100-240 VAC, 10 A Household outlet
Internal Power Unit	24-30v DC
Effective Continuous Operation	Optional - 25 min on Internal Source (UPS)
	Virtually limitless on External Power
**System Communication & Interface**	
RF Sensor (Radar)	Power Over Ethernet (POE)
Thermal Camera	Power Over Ethernet (POE)
**Operation**	
Operation Concept	Seating (0.7m -0.9m from RF Sensor)
Manual	User manual in English
**Display**	
Numeric Values	Heart Rate (BPM)<br>Respiration Rate (RPM)<br>Respiration Ratio (IE)<br>Temperature (C®)<br>Min / Max / Average readings
Graphs	Heart Rate (BPM)<br>Respiration Rate (RPM)<br>Respiration Ratio (IE)<br>Respiration Wave
**Measurement Parameters**	
Heart Rate (BPM)	40-150
Respiration Rate (RPM)	0-60
Respiration Ratio (IE)	-5 -+4
Respiration Wave	Configurable
Temperature	33 – 42 degrees Celsius
Test Duration (Configurable)	30-120 seconds
**Measurement Accuracy**	
Heart Rate (BPM)	+/- 5
Respiration Rate (RPM)	+/- 3
**Certifications**	
RF Sensor (Radar)	CE Safety certificate
Thermal Camera	CE & FDA
**RF Sensor (Radar) Parameters**	
Frequency Band	24.0 – 24.25 GHz
Emitting Power with Antenna	26 dBm
**General TAMAR Specifications**	
Weight	Weight 112kg / 247Lbs
Dimensions	L 135cm x H 90cm x W 90cm<br>(L 4.4ft x H 2.96ft x W 2.96ft)
Operating Temp	10 to +35 °C<br>(No direct sunlight or rain)
